# An extensive dataset of eye movements during viewing of complex images

**DOI:** 10.1038/sdata.2016.126

**Published:** 2017-01-31

**Authors:** Niklas Wilming, Selim Onat, José P. Ossandón, Alper Açık, Tim C. Kietzmann, Kai Kaspar, Ricardo R. Gameiro, Alexandra Vormberg, Peter König

**Affiliations:** 1 Institute of Cognitive Science, University of Osnabrück, 49069 Osnabrück, Germany; 2 Department of Neurophysiology and Pathophysiology, University Medical Center Hamburg-Eppendorf, 20246 Hamburg, Germany; 3 Department of Systems Neuroscience, University Medical Center Hamburg-Eppendorf, 20246 Hamburg, Germany; 4 Biological Psychology & Neuropsychology, University of Hamburg, 20146 Hamburg, Germany; 5 Department of Psychology, Ozyegin University, 34716 Istanbul, Turkey; 6 Medical Research Council, Cognition and Brain Sciences Unit, Cambridge CB2 7EF, UK; 7 Department of Psychology, University of Cologne, 50931 Cologne, Germany; 8 Frankfurt Institute for Advanced Studies (FIAS), 60438 Frankfurt/Main, Germany; 9 Ernst Strüngmann Institute (ESI) for Neuroscience in Cooperation with Max Planck Society, 60528 Frankfurt/Main, Germany

**Keywords:** Attention, Computational neuroscience

## Abstract

We present a dataset of free-viewing eye-movement recordings that contains more than 2.7 million fixation locations from 949 observers on more than 1000 images from different categories. This dataset aggregates and harmonizes data from 23 different studies conducted at the Institute of Cognitive Science at Osnabrück University and the University Medical Center in Hamburg-Eppendorf. Trained personnel recorded all studies under standard conditions with homogeneous equipment and parameter settings. All studies allowed for free eye-movements, and differed in the age range of participants (~7–80 years), stimulus sizes, stimulus modifications (phase scrambled, spatial filtering, mirrored), and stimuli categories (natural and urban scenes, web sites, fractal, pink-noise, and ambiguous artistic figures). The size and variability of viewing behavior within this dataset presents a strong opportunity for evaluating and comparing computational models of overt attention, and furthermore, for thoroughly quantifying strategies of viewing behavior. This also makes the dataset a good starting point for investigating whether viewing strategies change in patient groups.

## Background & Summary

By moving our eyes in fast and ballistic movements our oculomotor system constantly selects which parts of the environment are processed with high-acuity vision. The study of this selection process spans several levels of neuroscientific analysis because it requires relating behavioral models of viewing behavior to the activity of individual neurons and brain networks. One of the key challenges for understanding the neural basis of selecting saccade targets is therefore to establish behavioral models of viewing behavior. Such models depend on an appropriate task for sampling viewing behavior from observers. One natural possibility is free-viewing of pictures and other stimuli. We define free-viewing as a task that imposes no external constraints on what locations or parts of a stimulus should be looked at. Instead, what locations are interesting or rewarding are defined internally by the observer. The lack of external constraints has two important advantages. On the one hand, it naturally leads to a rich variety of viewing behavior across observers and stimulus categories that is nevertheless highly structured^1^. On the other hand, it implies that the task requires almost no training and undemanding instructions, such that it can easily be executed by children^2^, cognitively impaired individuals, and a variety of non-human species^3,4^. These properties make free-viewing ideally suited for the study of complex oculomotor control behavior.

Yet, because observers might select different viewing strategies, the analysis of free-viewing data requires data across many observers and stimuli. Presently, a number of datasets are publicly available. Specifically, this includes datasets that document viewing behavior of a rather small number of subjects on a large number of images^5,6^. However, studies combining a sizable set of stimuli and a larger number of subjects are sparse^7^. A more complete list of different contributions can be found at http://saliency.mit.edu/datasets.html. Here, we present a dataset of eye-movement recordings from 949 observers who freely viewed images from different categories to address this issue. We believe that this dataset will be a valuable resource for investigating behavioral and neural models of oculomotor control. First, computational modeling of viewing behavior is a challenging research field that depends on a gold standard for model evaluation and comparison. With 2.7 million fixations, the presented dataset will significantly increase the size of the corpus of available eye tracking data. Second, the size of this dataset allows fine-grained analysis of spatial and temporal characteristics of eye-movement behavior. This is an important aspect, since eye-movement trajectories are highly structured in space and time^8–11^, and increasing the temporal window of analysis requires increasing the amounts of data. Third, this dataset might act as a reference to identify changes in oculomotor control in specific subpopulations, e.g., after stroke or due to mental illness.

In summary, this unique dataset of viewing behavior will allow evaluations of models of viewing behavior against a large sample of observers and stimulus categories (Data Citation 1). In the following sections, we describe the origin of the contained data, detail pre-processing steps performed, and show how to use the overall dataset. We also give a short overview of basic properties of the dataset to allow other researchers to assess its usefulness for their own research questions.

## Methods

**Figure 1:**
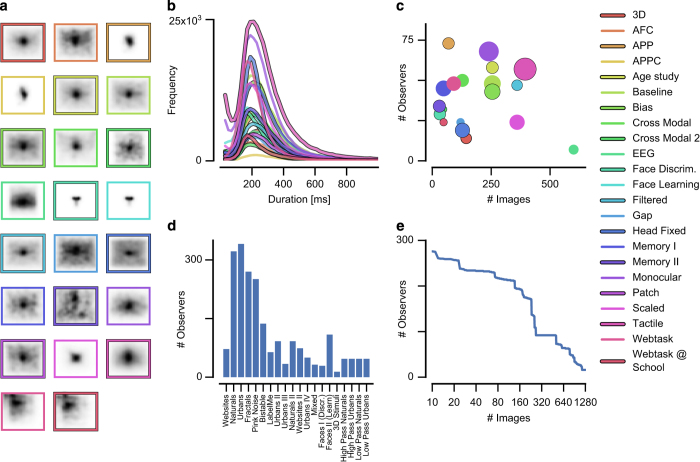
Dataset overview. (**a**) Smoothed spatial distribution of fixation locations for each study. The frame indicates screen borders. (**b**) Counts of fixation durations for each study. (**c**) A scatter plot showing number of observers and number of images per study. Circle size scales with the number of fixations, e.g. the difference between largest (35×10^3^ fixations) and smallest (1.3×10^3^ fixations) study is about a factor 27. (**d**) The number of observers per image set. (**e**) The plot shows how many images were seen by how many observers.

Our dataset contains about 2.7 million fixation locations from 949 observers, which viewed a total of 1,474 images (250 images each have fixations from more than 115 observers) from different image categories. The dataset aggregates data from 11 different published studies and adds 9 studies that have not yet been published. The main goal of this dataset is to combine these diverse studies and to harmonize their metadata to make them easily accessible for a larger audience. Tables 1 and 2 and Fig. 1 give an overview of the studies included in the dataset. The following paragraphs describe the general acquisition procedure that is common throughout the dataset.

Gaze coordinates were acquired with either a head mounted Eyelink II or remote EyeLink 1000 eye tracking system (SR Research Ltd., Ottawa, Ontario, Canada), sampled monocularly at 500 Hz. Operators of the gaze tracking system participated in a standardized training course before conducting a study, and thereby followed the same recording procedures (a detailed description is included in the dataset and available online at http://cogsci.uni-osnabrueck.de/~nbp/EyeTrackingInstruction.html). Accuracy of the gaze tracking system was checked with calibration and validation sessions before data recording. A general guideline for all recordings was to achieve an average validation error below 0.5° and to keep the maximal error below 1°. Studies that used the head mounted Eyelink II system additionally carried out repeated drift correction trials to compensate for slip of the eye tracker. The experimenter repeated calibration and validation sessions after breaks and whenever the drift correction error surpassed a predetermined threshold (usually >1° error). Participants removed any eye make-up before recording sessions to facilitate gaze tracking accuracy. Both systems were able to cope with most types of glasses and contact lenses. All participants had normal or corrected-to-normal visual acuity and were naïve to the purpose of the study. All studies were approved by either the ethics committee of the University of Osnabrück or the ethics committee oft the chamber of physicians in Hamburg. All participants gave written and informed consent before the start of the study. They were compensated monetarily (usually 5€/h) or in the form of course credits.

The eye tracking systems were capable of recording gaze location at high temporal frequency. They automatically generated fixation location and times from the raw gaze location time series, which were stored in the datasets. All studies used the SR-research default system parameters to define saccades: an acceleration threshold of 8000° per sec^2^, a velocity threshold of 30° per sec, and a deflection threshold of 0.1°. Fixations were defined as time periods without saccades. The dataset therefore consists of (x,y) gaze location entries for individual fixations. Coordinates were given in pixels with respect to the monitor coordinates (the upper left corner of the screen was (0,0) and down/right was positive). In many cases we also provide raw sample based data that can be used to validate fixation detection settings. Fixations were labeled with a subject ID, start and end times, image category and image number, the ordinal rank of the fixation within a trial (see Table 3 (available online only)), the trial within an experimental session, and a dataset ID that refers to the source study. Each study might define additional information for a fixation, such as experimental condition and subject specific information (see Table 3 (available online only)).

During construction of the dataset, we harmonized file and category names across studies to ensure that stimulus and category indices referred to the same stimuli. An important consequence of this harmonization was that the dataset contained stimuli in their original size only. Since stimuli might have been presented on different displays with different resolutions and sizes, the user of the dataset has to transform the gaze locations to match the original stimulus or to rescale the stimuli to the size used during presentation. Table 2 gives stimulus sizes, display resolution (in pixel and degree), stimulus position on the screen, viewing distance, and pixels per degree.

The dataset contains anonymised data, where a numerical ID identifies studies and participants. No personal information is contained.

**Figure 2:**
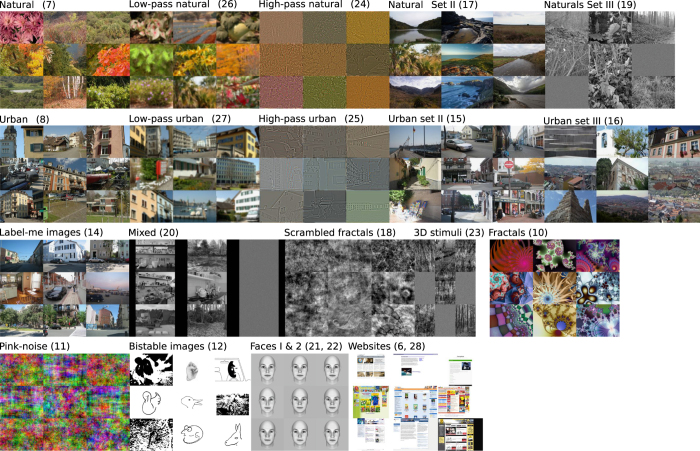
Image category overview. Each panel shows nine example images from a different category.

The following paragraphs provide more information about the individual studies. Examples of stimuli are provided in Fig. 2.

### Baseline [ID 3, 48 participants, 20.3×10^4^ fixations]

This study^12^ investigated eye-movements of 48 participants during free-viewing of 255 different images in 4 different categories (Natural, Urban, Fractal, Pink noise). Subjects were instructed to study the images carefully. Images were presented for 6s in a randomized order. Stimuli used in the Age, AFC, Bias, Gap, Filtered, Head Fixed, Memory I, Monocular, Patch, and Tactile studies were based on the stimulus set used in this study.

### AFC [ID 2, 20 participants, 3.9×10^4^ fixations]

The stimuli were either unmodified fractals or globally and/or locally modified images derived from the same fractals. Global modifications concerned the addition of varying degrees of noise to the phase spectrum of the fractals. Local modifications entailed local increases or decreases in luminance contrast at five locations. The viewing duration was 5 s. After exploration of the stimuli, participants performed a recognition task. The same stimulus was shown together with the unmodified or another luminance modified version of the stimulus. The observer’s task was to identify the one they had just seen.

### Age study [ID 0, 58 participants, 10.5×10^4^ fixations]

This is a patch recognition experiment that compares viewing behavior of three different age groups (school children, students, and elderly)^2^. Participants saw 64 images from the categories Natural, Urban, and Fractal, and 63 images from the category Pink-noise. Stimulus presentation was balanced such that pairs of observers saw all images within each of these four categories (255 in total). The presentation time was 5 s.

### Bias [ID 11, 43 participants, 17.6x10^4^ fixations]

This study^13^ investigated the occurrence of horizontal biases during free-viewing. It corresponds to the data of the first experiment in ref. 13. Only right-handers participated in this experiment. Participants viewed 255 images from the Natural, Urban, Fractal, and Pink Noise categories for 6 s each. Subjects explored a mixture of original and mirror-reversed versions of the images. Each subject explored only one version, original or reversed, of each image.

### Filtered [ID 21, 47 participants, 8.3x10^4^ fixations]

This study^13^ investigated the influence of handedness and spectral content on the occurrence of horizontal biases during free-viewing. It corresponds to the data of the second experiment in ref. 13. The experiment consisted of 31 right-handers and 17 left-handers. Participants viewed 120 images for 6 s each. Each image was preceded by a drift correction. Images were presented either in an original or mirror-reversed version, and either with full spectral content or low-pass or high-pass filtered (Gaussian filter, cutoff of 0.6 c/degree). Each subject explored only one version of each image. In half of the trials, the drift correction fixation dot remained visible for 1s after the stimulus onset, and we requested subjects to keep fixating until it disappeared (delay trial). If a subject’s gaze moved away from a radius of 1 visual degree from the center, the trial terminated, and a feedback message was delivered. Delay and non-delay trials were blocked across the experiment.

### Gap [ID 22, 24 participants, 4.9x10^4^ fixations]

This study^13^ investigated the influence of drift-correction trials on horizontal biases during free-viewing. It corresponds to the data of the third experiment in ref. 14. 24 right-handers participated in the experiment. Participants viewed 120 images for 6s each. We introduced temporal gaps of 0, 300, 600, and 900 ms between the disappearance of the fixation dot in the middle of the screen for drift-correction and the appearance of the images. During the temporal gap, the screen was at the gray scale level of the drift-correction period, and the gap duration was randomized across trials. Subjects did not receive any instruction in relation to the existence of a gap.

### Head Fixed [ID 15, 19 participants, 15.1×10^4^ fixations]

This study investigated whether head restraints might alter saccade target selection. Participants freely viewed 64 images from the urban and fractal categories each for 6s recorded in a head fixed (1) and head free (2) condition. In the head fixed condition, participants placed their chin on a chin rest and additionally bit into a mouth guard fit for each participant. Stimuli were randomized, such that pairs of observers saw all images in all conditions, i.e., each observer saw 32 images from a category in the head fixed and 32 in the head free condition. The study also contained a guided viewing task where observers had to follow a point which jumped to a new location once it was fixated. In this experiment, the average validation error did not surpass 0.55° with the exception of subject 6 (0.74° in condition 1).

### Memory I [ID 4, 45 participants, 17.9×10^4^ fixations]

Participants freely observed 48 images in a randomized order and with five repetitions^15,16^. They consecutively saw 5 blocks of all images. The block number is coded as ‘iteration’. The images equally covered four categories, namely Natural, Urban, Fractal, and Pink noise images. Presentation duration was 6s for each image. Before an image appeared, participants had to fixate on a cross presented in the center of the screen. A short 5 minute break after the third presentation block maintained participants’ alertness and avoided potential fatigue.

### Memory II [ID 5, 34 participants, 10.9×10^4^ fixations]

The design of this study^15^ was similar to that of Memory I with exceptions noted. Participants repeatedly explored 30 urban images for 6s each. The images differed regarding their complexity and were grouped in 5 consecutive blocks. Ten images depicted global scenes containing many houses, streets, and other objects (high complexity); 10 images depicted local arrangements such as single houses (medium complexity); 10 images depicted close-ups of urban details, such as park benches or staircases (low complexity). Four independent raters judged image complexity and showed a perfect inter-rater agreement. A high image resolution (2560x1600px) conserved details for an in-depth exploration. After the experiment ended, participants once more observed all images for 6s. However, this time they were asked to explore those image regions that they considered uninteresting. We conducted this additional trial for exploratory reasons. The corresponding data have not been included in the published results but are included here (iteration=6).

### Monocular [ID 12, 68 participants, 31.4×10^4^ fixations]

This unpublished study investigates the occurrence of viewing biases in monocular vision. All participants viewed the images with their right eye, the left eye was occluded with an eye patch. Participants freely observed 240 images for 6s each. All images were shown at 30′′, some images were resized by bicubic interpolation to the corresponding ratio and resolution of 2560×1600.

### Patch [ID 1, 35 participants, 5.6×10^4^ fixations]

This recognition experiment presented fractals with local contrast modifications and phase scrambling. The base stimuli were identical to the AFC study. Participants explored stimuli for 5 s. Subsequently, the participants indicated whether a local image patch, taken from the previous or a randomly selected stimulus, originated in the previously explored stimulus or not.

### Tactile [ID 8, 57 participants, 35.8×10^4^ fixations]

This study^17^ evaluated the effect of task irrelevant tactile stimulation on free-viewing of images. Participants placed their hands on a table in front of them either in a crossed or uncrossed posture. Subjects received stimulation over the back of their hand, either to the right, the left, or both hands, at random times during image exploration. Images were presented in 16 blocks of 24 images each. Eye tracker drift-correction preceded the first image of each block. Participants altered their hand posture every 4 blocks. Each image was presented for at least 6s. The appearance of the next image was contingent upon subjects’ gaze position. Specifically, the presentation switched to the next image after a fixation had begun in an area inside 6° around the images’ vertical meridian. In half of the trials, tactile stimulation occurred 150 ms after an image change. In the other, tactile stimulation took place randomly at any moment between 0.5 s and 6 s.

### 3D [ID 20, 14 participants, 8.5×10^4^ fixations]

This study^18^ investigated visual exploration of natural images under stereoscopic presentation conditions using specialized equipment. 3d images of natural scenes were taken using a pair of digital cameras. These photographed scenes were also laser-scanned to obtain the ground-truth depth structure of the scenes. These depth-maps allowed presentation of the depth structure independent of image content and therefore made it possible to study the influence of binocular disparity information on eye-movements. Each image was presented either stereoscopically (3d) or not (2d). Furthermore a given depth map was presented either with its corresponding luminance information (natural), or following spectral modifications (pink noise or white noise), leading to 6 conditions across 2 factors. Presentation duration was 20 s. Participants were required to press a button as soon as they recognized at least two depth layers in the images.

### Cross Modal [ID 16, 29 participants, 12×10^4^ fixations]

This study^19^ investigated how visual and auditory sources of information were integrated during free-viewing of natural images, and 64 natural images were shown, either presented from the left or right side of the monitor (Audio-visual conditions, AVL or AVR) or without any sounds (Visual condition). Sounds were played during the presentation of visual stimuli through speakers flanking the monitor. Presentation time was 6s. Auditory stimuli consisted of natural sounds (e.g., bird sounds). During the auditory condition, sounds were played while white noise images were presented. Subjects were instructed to study the images and listen to the sounds carefully.

### Cross Modal 2 [ID 17, 32 participants, 3.1×10^4^ fixations]

This study^20^ extended the Cross Modal study^19^ to 4 different sound locations. Auditory stimuli were presented through in-ear binaural earphones and spatial localization of stimuli was achieved using a software-based solution. Stimulus duration was 4s. A total of 9 different conditions (4 audio, 4 audiovisual, and 1 visual) were presented across 96 trials (24 visual, 24 auditory, and 12×4 audiovisual trials).

### EEG [ID 9, 7 participants, 7×10^4^ fixations]

This unpublished study investigates the electroencephalographic correlates of free-viewing exploration. After approximately one-hour preparation time for the EEG recording, subjects explored 150 landscapes and urban images for 8 s each, in blocks of 30 trials. Subjects performed this task in three or four different sessions on different days, resulting in the exploration of 450 or 600 different images per subject.

### Scaled [ID 10, 24 participants, 16.6×10^4^ fixations]

This study was designed to investigate exploration and exploitation on stimuli with varying spatial properties. Participants freely observed 360 images from the categories urban, nature, and webpages for 6s each. The images were presented in five different sizes (7′′, 10′′, 15′′, 21′′, and 30′′). The 30′′ images served as the full size condition. The remaining sizes were achieved by either scaling down the image coordinates from 30′′ to the desired size or by cropping out the central part of the 30′′ image according to the desired size. The field ‘scaled’ indicates whether a stimulus was scaled or cropped. The background color for smaller images was set to neutral gray (RGB color: 128, 128, 128).

### Webtask [ID 13, 48 participants, 15.1×10^4^ fixations]

Participants saw screenshots of 90 websites in three different task conditions^21^. Stimulus presentation was balanced such that triplets of observers saw all stimuli in all conditions. The first task was a free-viewing task in which participants were instructed to ‘simply explore the website’ for 6 s. The second task, the content awareness task, was similar, but participants had to select a target user group for each site afterwards. The third task presented a search term before stimulus presentation and participants had to rate how well the website fit to the search term. The dataset contains fields that encode the user group rating, the shown user groups, the relevance of the search term, and a familiarity rating of the website.

### Webtask @ School [ID 14, 24 participants, 4.0×10^4^ fixations]

This study is similar to the webtask study. A subset of 60 webtask stimuli was shown to school-children attending 6th grade in a secondary school in a small town in Germany. All other aspects were equal to the webtask study.

### APP [ID 6, 73 participants, 9.9×10^4^ fixations]

This study^14^ investigated eye-movements leading up to and following the initial perception of ambiguous and disambiguated line drawings. Data from 73 naïve participants were included. They viewed 11 ambiguous stimulus sets, each including an ambiguous and two disambiguated stimuli, as well as 36 control stimuli. Participants freely explored the images in order to identify what was shown. They pressed a button upon successful recognition. Following the button press, the stimuli remained visible for another 4 s. Afterwards, participants indicated prior knowledge of the stimulus and rated their perceptual certainty.

### APPC [ID 7, 46 participants, 1.2×10^4^ fixations]

Similar to APP above, participants freely explored line drawings with the goal of identifying the content^22^. Contrary to APP, this paradigm placed the drawings in context. These were congruent with one of the two interpretations of the ambiguous stimulus. Triggered by the first saccade, the context was immediately taken off screen, and the experiment then followed the procedures in APP. Eight ambiguous and disambiguated stimulus sets were included, as well as eight unambiguous control stimuli. Data from 46 participants were included in the dataset.

### Face Discrimination [ID 18, 29 participants, 10.0×10^4^ fixations]

This study investigated eye-movements during a face discrimination task. Faces were computer-generated to form a circular similarity continuum spanning 360 degrees in steps of 11.25 degrees (32 faces). Participants were randomly associated with a pair of opposing faces (separated by 180 degrees, labeled 0 or 180). In each trial one of the reference faces was shown (duration: 1.5 s) together with a different test face. Participants reported at the end of the trial whether the two faces were the same or different. Depending on the performance of the participant, an adaptive algorithm decided on the angular distance between the reference and test faces for the next trial (for example: 0 degrees *vs* 22.5 degrees or 180 degrees *vs* 168.75 degrees). Two psychometric functions, mapping angular distances to the probability of perceiving a difference, for the two reference faces were derived. The same discrimination task was repeated following a learning procedure (see Face Learning below), which required participants to associate an aversive outcome with one of the faces. Stimuli spanned 27° to approximate face sizes during everyday interactions.

### Face Learning [ID 19, 104 participants, 14.5×10^4^ fixations]

This study tested the effect of aversive associative learning on the exploration of faces. Eight faces, separated by 45 degrees, were selected for this experiment (see Face Discrimination above). During the conditioning phase, one randomly selected face was paired with an aversive outcome (mild noxious stimulation of one hand in 33% of trials), whereas the most dissimilar face (separated by 180 degrees) was kept neutral. Following this learning phase, all faces were presented and the effect of aversive learning on the exploration of faces was investigated. Before aversive learning (baseline phase), faces were all neutral, and the aversive stimuli were delivered in a predictable manner following a non-face symbol. As in the face discrimination task, stimulus duration was 1.5 s. Subjects were required to press a button as soon as an oddball target (blurred face) was presented. Before and after the aversive learning, some participants performed a perceptual discrimination experiment (see Face Discrimination above).

### Code availability

We provide python and MATLAB code to load the dataset. Python code was tested with python 2.7, h5py version 2.5.0 and HDF5 version 1.8.15. We tested MATLAB code with version 8.3.0.532 (R2014a). This code is distributed with the dataset and subject to the same license.

## Data Records

The dataset consists of one HDF5 file (‘etdb_1.0.hdf5’), which contains eye tracking data, a folder that contains stimuli (‘Stimuli’) and one semicolon-separated text file (‘meta.csv’, semicolon-separated file with UTF-8 encoding) that contains experimental metadata associated with each individual dataset (Data Citation 1).

The file ‘etdb_1.0.hdf5’ is a standard HDF5 file created with h5py version 2.5.0 and HDF5 version 1.8.15. HDF5 allows the structuring of data into groups much like a file system organizes data with folders and files. In this case, each study in the dataset is stored in a group whose name corresponds to the study name. Within each group, we store vectors that encode information about fixations. Each index of these vectors encodes a fixation, i.e., accessing etdb_1.0.hdf5 at AFC/x[10] retrieves the horizontal location of the tenth fixation in the AFC study. Table 3 (available online only) shows what information is encoded for every study in ‘etdb_1.0.hdf5′. Some experiments require additional information to correctly interpret data from a trial. For example, the webtask study presented search terms, potential user groups, and URLs in some of the trials. This information is represented for each fixation by a linear index into an attribute of a group. For example, if the ‘url’ field ‘etdb_1.0.hdf5/Webtask/url[5]’ is 2, then the corresponding url is encoded in ‘etdb_1.0.hdf5/Webtask/attrs/url[2]’. This index is 1-based, i.e., 1 refers to the first element in an attribute list.

The file ‘meta.csv’ is a csv file with a table that contains meta-information about each study. In particular, it contains stimulus sizes, display sizes (in pixel and degree), and a conversion factor to translate pixels to degrees of visual angles. This allows mapping fixation locations onto stimuli.

The stimuli are located in ‘Stimuli/’, which contains subfolders for each stimulus set. Stimulus sets are encoded numerically (6—Websites, 7—Natural, 8—Urban, 10—Fractal, 11—Pink noise, 12—APPC bistable image set, 14—LabelMe images, 15—Urban set II, 16—Urban set III, 17—Natural set II, 18—Scrambled fractals, 19—Natural set III, 20—Mixed, 21—Faces I (Discr.), 22—Faces II (Learn), 23—3D Stimuli, 24—High Pass Natural, 25—High Pass Urban, 26—Low Pass Natural, 27—Low Pass Urban, 28—Websites set II). Within each category folder stimuli are numbered. Fixations can be mapped according to their ‘category’ and ‘filenumber’ fields, i.e., category 8 and filenumber 11 map to the path ‘Stimuli/8/11.{png,bmp,jpg}’.

Unfortunately we were not able to obtain the rights to publish four of the 64 fractal stimuli in category 10 under a CC0 license. Some of these were obtained from fractal collections on the internet whose authors we were unable to contact. However, we made sure that all fractals are free of use for research purposes. We can provide these stimuli upon request.

We also distribute additional raw data files and metadata wherever available. Metadata is distributed as comma separated text files that map subject IDs to metadata. Each file contains descriptions of the respective columns. These files can be found in the folder ‘additional_metadata/’. Sample based data is provided, wherever possible, as additional HDF files with a similar structure ‘etdb_1.0.hdf5’. Instead of fixations each vector here contains x,y locations of each sample provided by the eye tracker. Field names are the same as in the fixation based dataset. Sometimes fields will be prefixed by ‘left’ or ‘right’ to distinguish which eye was tracked. In this case x,y positions are encoded in fields called ‘left_g{x,y}’ or ‘right_g{x,y}’. Sample based data files can be found in the folder ‘additional_samples/’.

## Technical Validation

One of the most important aspects of the reliability of gaze-tracking is its spatial accuracy. The data in this dataset were recorded with two high precision eye trackers (Eyelink II and Eyelink 1000) that are known for their high accuracy. Furthermore, a calibration and validation session preceded every recording block and data recording was only started when the average error fell below a pre-specified threshold. The threshold depends on the study (Table 2), but is always smaller than 0.6° of the visual angle. Studies that used the head mounted Eyelink II system frequently checked tracking accuracy by presenting drift correction trials. In these trials, participants fixate on a dot, which allows calculating the measurement error of the tracking system.

A second important aspect of reliability is the temporal accuracy of saccade onsets and offsets. Data in this dataset were sampled at 250 or 500 Hz, which is very fast in relation to fixation durations (200–300 ms). Figure 1b shows a histogram of fixation durations for all contained studies.

A final consideration is the proficiency of users that operate eye tracking equipment. A standardized training system ensured proficiency. It teaches all new users how to operate the equipment and how to deal with common difficulties (e.g., make-up, glasses, etc.). Users at the University-Medical Center in Hamburg-Eppendorf all underwent the same training procedure.

## Usage Notes

This dataset is distributed in open and standardized file formats (HDF5, text, PNG) and can therefore be processed with many software packages. In particular, we made sure that the data can easily be read with python, R, and MATLAB.

Users should keep in mind the following caveats. First, the duration of a fixation is encoded by its end—start time. Please note that the end and start time themselves are meaningless, since they are expressed relative to some unknown point within the experiment. Second, mapping fixations to stimulus locations requires either mapping gaze locations onto the stimulus or scaling the stimulus appropriately. For example, the stimuli in the ‘Head Fixed’ study were shown on a screen with a 16:9 aspect ratio while the images were 4:3. This leaves a gray border of 240px to the left and right of the image, which are not included in the image file in the dataset. Horizontal (x) coordinates smaller than 240 pixels and larger than 1680 pixels are therefore outside the image. Third, in most cases participants had to fixate a fixation dot before stimulus onset, and the first fixation within a trial can be driven by this fixation dot (in some studies fixation onset times <0 are indicative of this). In some cases, the fixation dot remained visible for a while after an image change, or there was a gap between disappearance of the dot and appearance of the image. In these cases, the trial 0 time corresponds to the onset of the image or of the gap period. Fourth, in some experiments, images were presented in their original and mirrored versions. Since images were provided only in their original versions, these images need to be left-right flipped when mapping gaze coordinates from mirrored trials to images.

## Additional information

**How to cite this article**: Wilming, N. *et al.* An extensive dataset of eye movements during viewing of complex images. *Sci. Data* 4:160126 doi: 10.1038/sdata.2016.126 (2017).

**Publisher’s note**: Springer Nature remains neutral with regard to jurisdictional claims in published maps and institutional affiliations.

## Supplementary Material



## Figures and Tables

**Table 1:** Studies and associated meta data.

**Name**	**ID**	**Article**	**Categories**	**Task**	**V. Dur.**	**# Obs.**	**# Fix.**	**Age**
3D	20	18	23	Depth rec.	20s	14	84093	Students
AFC	2		18	Image rec.	5s	20	39358	Students
Age study	0	2	7, 8, 10,11	Patch rec.	5s	58	105813	7.6, 22.1, 80.6
APP	6	14	12	Object rec.	Var.	73	99101	25.6 (SD 8.9)
APPC	7	22	12	Object rec.	Var.	46	12866	25 (SD 6)
Baseline	3	12	7, 8, 10, 11	FV	6s	48	203772	23.1 (19–28)
Bias	11	13	7, 8, 10, 11	FV	6s	43	176391	23.1 (19–28)
Cross Modal	16	19	19	FV	6s	50	120261	Students (23)
Cross Modal 2	17	20	20	FV	6s	32	31826	Students (19–36)
EEG	9		14	FV	8s	7	70026	Students
Face Discrim.	18		21	Discrimin.	1.5s	29	100448	26.6 (SD 4.54, 19–35)
Face Learning	19		22	Aversive learning	1.5s	104	145378	26.9 (SD 4.14, 20–36)
Filtered	21	13	7, 8, 24, 25, 26, 27	FV	6s	47	83834	22.6 (SD 2.3, 19–28)
Gap	22	13	7, 8	FV	6s	24	49208	22 (SD 2.5, 19–28)
Head Fixed	15		8, 10	FV, GV	6s	19	151338	24 (18–41)
Memory I	4	15, 16	7, 8, 10, 11	FV	6s	45	179473	24.2 (18–48)
Memory II	5	19, 15	16	FV	6s	34	109830	25.9 (19–49)
Monocular	12		15, 17	FV	6s	68	282602	22.4 (SD 2.98)
Patch	1		18	Patch rec.	5s	35	64449	Students
Scaled	10		15, 17, 18	FV	6s	24	166156	21.38 (+/- 3.00)
Tactile	8	17	7, 8, 10, 11, 14	FV	min 6s	57	358578	21.8 (18–29)
Webtask	13	21	6	FV, CAT, IST	6–12s	48	151581	41.5 (24–55)
Webtask @ School	14		6	FV, CAT, IST	6–12s	24	40553	12 (+/- 0.3)
‘V. Dur.’=Viewing duration, ‘# Obs.’=number of observers, ‘# Fix.’=number of fixations, ‘rec.’=recognition, ‘Discrimin.’=discrimination, ‘FV’=free-viewing, ‘GV’=guided viewing, ‘CAT’=content awareness task, ‘IST’=information search task. ‘Students’ implies that participants were recruited from the student population at the University of Osnabrück but that no further age information is available.								

**Table 2:** Stimulus presentation and recording information metadata.

**Name**	**Display**	**Display resolution (pixels)**	**Disp. size (degree)**	**Img. size (pixel)**	**Img. pos. (pixel)**	**V. dist. (cm)**	**PPD**	**Sampling freq. (Hz)**	**Val. error (degrees)**	**Eye Tracker**
3D	SeeReal C-s	640×1024	30.9×24.9	1280×1024	0	65	19.1	250 Hz	0.3	EL II
AFC	ViewSonic	1280×960	36.2×27.2	1280×960	0	65	35.3	250 Hz	0.3	EL II
Age study	ViewSonic	1280×960	35 × 26.5	1280×960	0	65	36.3	500 Hz	0.6	EL 1000
APP	Apple Cinema	2560×1600	23.8×23.8	variable	central	60	41.6	500 Hz	0.3	EL II
APPC	Apple Cinema	2560×1600	23.8×23.8	variable	central	60	41.6	500 Hz	0.3	EL II
Baseline	SM1100	1280×960	29×22	1280×960	0	80	45.6	500 Hz	0.3	EL II
Bias	SM1100	1280×960	28×21	1280×960	0	80	45.6	500 Hz	0.3	EL II
Cross Modal	SM1100	1024×768	28×21	1024×768	0	80	36.5	500 Hz	0.3	EL II
Cross Modal 2	Apple Cinema	2560×1600	44×36	1944×1600	308, 0	60	58	500Hz	0.5	EL II
EEG	U2311Hb	1920×1080	27×46	1280×960	320, 6	60	41	500 Hz	0.5	EL 1000
Face Discrimin.	SM 204B	1600×1200	40.6×30.5	1600×1200	central	50	39	250 Hz	0.3	EL 1000
Face Learning	SM 204B	1600×1200	40.6×30.5	1600×1200	central	50	39	250 Hz	0.3	EL 1000
Filtered	SM1100	1280×960	28×21	1280×960	0	80	45.6	500 Hz	0.3	EL II
Gap	SM1100	1280×960	28×21	1280×960	0	80	45.6	500 Hz	0.3	EL II
Head Fixed	BenQ XL2420T	1920×1080	53.8×30.24	1440×1080	240,0	60	38	500 Hz	0.55	EL 1000
Memory I	SM1100	1280×960	29×22	1280×960	0	80	45.6	500 Hz	0.35	EL II
Memory II	Apple Cinema	2650×1600	46.2×28.9	2650×1600	0	80	55.4	500Hz	0.35	EL II
Monocular	Apple Cinema	2560×1600	46.2×28.9	variable	0	80	55.4	500Hz	0.5	EL II
Patch	ViewSonic	1280×960	36.2×27.2	1280×960	0	65	35.3	250Hz	0.3	EL II
Scaled	Apple Cinema	2560×1600	46.2×28.9	640×400–2560×1600	central	80	55.4	500Hz	0.3	EL II
Tactile	SM1100	1280×960	28×21	1280×960	0	80	45.6	500Hz	0.5	EL II
Webtask	SyncMaster 971p	1280×1024	35.56×28.4	1272×922	4,86	60	36	500Hz	0.3	EL II
Webtask @ School	SyncMaster 971p	1280×1024	35.56×28.4	1272×922	4,86	60	36	500Hz	0.5	EL 1000
‘Disp. size’=display size, ‘Img. size’=image / stimulus size in pixel, ‘Img. pos (pixel)’=upper left corner of the image (x,y=0,0=top left of screen; ‘central’=each stimulus was centered on the screen; y=0 is omitted), ‘V. dist.’=viewing distance measured between eyes and screen, ‘PPD’=pixels per degree of visual angle, ‘Sampling freq’=sampling frequency of gaze position, ‘Val. error’=maximal average validation error before gaze tracking started, ‘EL II’=Eye Link II, ‘EL 1000’=Eye Link 1000.										

**Table 3:** Description of fields in the dataset

**Dataset**	**Fieldname**	**Description**
**All**	*SUBJECTINDEX*	Subject ID
	*dataset_nr*	Dataset
	*x*	X-position of fixation
	*y*	Y-position of fixation
	*start*	Start of fixation in ms
	*end*	End of fixation in ms
	*category*	Category Nr.
	*filenumber*	File number of viewed image
	*fix*	Fixation number within trial
	*trial*	Trial # within experiment
**Tactile**	*blockstart*	1 f the trial was the start of a block
	*mirror*	1 if image is left-right mirrored
	*block*	1 if hands crossed
	*stim*	stimulation at trial start (at 150ms, 1:left; 2:right; 3:bilateral)
	*latestim*	1 if stimulation occurred later during the trial
	*value*	Side of late stimulation (1: left; 2:right; 3:bilateral)
	*stim_time*	time of late stimulation
	*block*	Training trials=0, Experimental trials=1
**APPC**	*buttonpress1*	Time of button press upon recognition of shown object
	*buttonpress2*	Time of button press in cases of flipped perception
	*certainty*	Rating of perceptual certainty
	*context*	Code for context version shown
	*context_viewtime*	Time until first saccade away from drift position
	*known*	Prior knowledge of the Stimuli
	*mod*	1=ambiguous, 2,3 unambiguous
	*percept1*	Code for initial perception
	*percept2*	Code for potential second percept
**AGE**	*age*	Age group of the participant (Children: 0, Younger Adults: 1; Older Adults: 2)
	*answer*	Answer (0: No; 1: Yes)
	*catch*	Is the trial a catch trial? (0: No; 1: Yes)
	*patchpos*	The central location of the patch in the image from which it is taken
	*valid*	Is the fixation location within the monitor (0: No, 1: Yes)
**AFC**	*mod*	The local luminance contrast modification weight
	*scr*	The amount of noise added to the phase spectrum
**Scaled**	*Block*	Block within session (In one block only pictures of one size were shown)
	*Category*	1=website, 2=urban, 3=landscape
	*Index*	Image index
	*scaled*	1=scaled image, 2=cropped image
	*session*	Each session showed 5 blocks/sizes. Break in between each session
	*size*	Presented image size
	*onscreen*	Fixation within monitor coordinates.
	*onpicture*	Fixation within image coordinates of respective size
**Patch**	*mod*	The local luminance contrast modification weight
	*scr*	The amount of noise added to the phase spectrum
	*image*	The base image used for modifications
**APP**	*buttonpress1*	Time of buttonpress upon recognition of shown object
	*buttonpress2*	Time of buttonpress in cases of flipped perception
	*certainty*	Rating of perceptual certainty
	*known*	Prior knowledge of the Stimuli
	*mod*	1=ambiguous, 2,3 unambiguous
	*percept1*	Code for initial perception
	*percept2*	Code for potential second percept
**Webtask & Webtask @**	*condition*	1: free viewing task, 2: information search task, 3: content awareness task.
**School**	*example*	1 if example trial.
	*familiarity*	Familiarity rating, ranges from 1 (never seen) to 5 (well known).
	*on_image*	1 if fixation was on the stimulus.
	*orig_cats*	Original category used in the article. 1:news, 2:blogs, 3:landing pages, 4:shops, 5:company, 6:information.
	*orig_filenumber*	Original filenumber within a category.
	*relevance*	Relevance during content awareness task, ranges from 1=not relevant to 5=highly relevant.
	*search_result*	Searchterm used during the information search task.
	*url*	URL displayed during this trial
	*user1-5*	User groups that the participant could choose from.
	*user_group*	Selected user group (1–5).
**Head Fixed**	*condition*	1: head fixed with chin rest and mouth guard, 2: head free, 3: break between head fixed and head free block, 4: break
	*guided_viewing*	1 for guided viewing trials
**Face**	*Phase*	Before (1) or after (5) the aversive learning
**Discrimin.**	*Isref*	Whether the trial is a reference face (0 or 180 degrees) or not.
	*chain*	Reference face identity. Chain 1 will be associated to an aversive outcome, whereas chain 2 will stay neutral (only face learning).
	*condition*	Angular distances (x100) from the reference faces.
**Face Learning**	*oddball*	1 if trial is oddball.
	*ucs*	1 if aversive outcome is delivered during the trial otherwise 0.
	*phase*	before (2), during (3), after (4) aversive learning
	*condition*	-135, -90, -45, 0, 45, 90, 135, 180: Angular distances from the face that will be associated to an aversive outcome. 500: Trials with aversive outcome 1000: oddball trials 3000: Null trials.
	*training*	1 if observer performed a perceptual discrimination task before.
**3D**	*image*	Scene number, 24 in total. A given scene is presented with or without depth information in 3 different versions: original, pink noise, white noise.
**Bias**	*mirror*	1 if image is left-right mirrored
**Filtered**	*handedness*	0—right-handers; 1—left-handers
	*spatial_filter*	1: no filter, 2: high pass filter, 3: low pass filter
	*mirror*	1 if mirrored
	*delay*	1 if delay
**Gap**	*mirror*	1 if image is left-right mirrored
	*gap*	Gap in ms between disappearance of drift correction dot and appearance of image (0,300,600,900)
**Memory I**	*iteration*	1=1st presentation of image; 2=2nd presentation; etc.
	*on image*	1=fixation on image; 0=fixation beyond image borders
**Memory II**	*iteration*	1=1st presentation of image; 2=2nd presentation; etc.
	*condition*	Complexity of image (1=low, 2=middle, 3=high)
	*on image*	1=fixation on image; 0=fixation beyond image borders
**Crossmodal2**	*condition*	10=visual; 11 => audio-visual (top-left); 12 => audio-visual (bottom-left); 13 => audio-visual (top-right); 14 => audio-visual (bottom-right); 21 => audio (top-left); 22 => audio (bottom-left); 23 => audio (top-right); 24 => audio (bottom-right)
**Crossmodal**	*condition*	1=visual; 2 => audio-visual left; 3 => audio-visual right; 4 => audio left; 5 => audio right
